# Solution-Phase Design of Emerging Nanomaterials

**DOI:** 10.1021/acs.chemmater.5c01948

**Published:** 2025-11-21

**Authors:** Florian M. Schenk, Maksym Yarema

**Affiliations:** Chemistry and Materials Design Group, Institute for Electronics, Department of Information Technology and Electrical Engineering, 27219 ETH Zurich, Zurich CH-8092, Switzerland

## Abstract

Emerging materials such as liquid metals, intermetallics, phase-change materials, and glassy chalcogenides provide unconventional properties to drive innovations in catalysis, biomedicine, photonics, and data processing. However, the same unique characteristics that enable these functionalities, such as dynamic surfaces, complex bonding, or amorphous structures, simultaneously present significant challenges in controlling their synthesis and tailoring them for specific applications. Solution-phase assembly of these materials from nanoscale building blocks offers a powerful avenue to overcome these barriers. This approach provides unprecedented nanoscale control and high flexibility to adjust composition, geometry, and surface, while also enabling advanced patterning technologies and providing compatibility with various substrates. This perspective highlights the transformative potential of solution-phase synthesis for the development of next-generation functional materials. More broadly, we showcase the avenue toward precise design of materials as well as accelerated materials discovery and optimization, particularly when integrated with modern machine learning-based algorithms. This has far-reaching implications, complementing and even replacing conventional fabrication methods as well as high-throughput materials screening and optimization.

## Graphical abstract

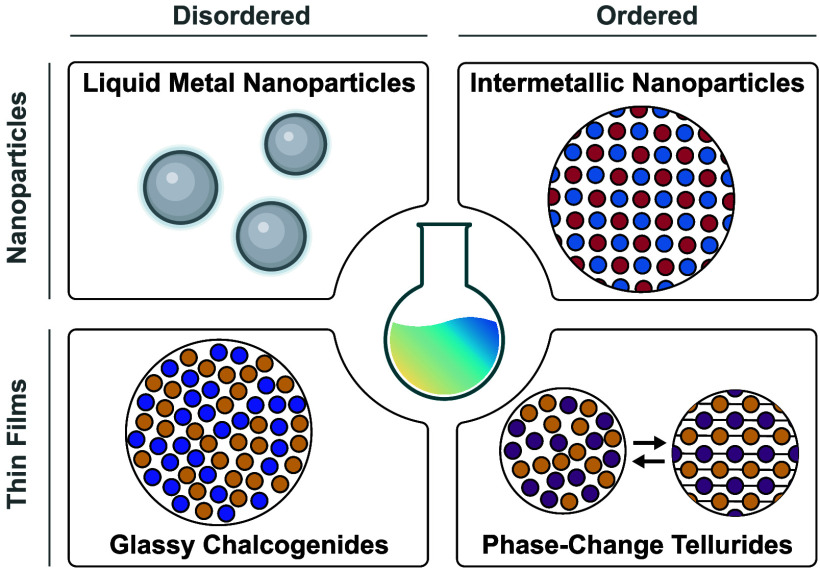

## 1 Introduction

Next-generation materials hold immense potential for innovations across diverse fields, including catalysis,^1^ biomedicine,^2^ photonics,^3^ data storage,^4^ and neuromorphic computing.^5^ Emerging materials classes, such as liquid metals, intermetallics, or phase-change chalcogenides, are particularly promising through their unique characteristics that enable rich functionalities, which range from unconventional bonding^6,7^ and metastable, amorphous or anisotropic structures,^8^ to dynamic surfaces.^9^ However, these very properties also present significant challenges in controlling these materials during their synthesis and device integration. Issues like mismatched reactivity,^10^ oxygen sensitivity,^11,12^ and metastable phases^13^ hinder the reliable fabrication of next-generation technologies. In addition to synthetic challenges, unlocking the full potential of these materials for specific applications requires precise tailoring of their elemental composition, crystal phase, assembly, morphology, and surface properties.

Solution-phase processing offers a powerful and versatile avenue to overcome some of these limitations. It leverages the assembly of nanoscale building blocks, from nanoparticles or molecular clusters, at ambient temperature and pressure, providing unprecedented control over material properties on the nanoscale: Flexible tuning of composition is enabled through multicomponent nanoparticle synthesis^14^ or direct mixing of different molecular clusters.^15^ Geometric versatility is provided by diverse deposition techniques, from spin-coated thin films to 3D-printed structures. Crucially, solution processing is also compatible with a wide variety of rigid, flexible, or prepatterned substrates. Beyond that, liquid-phase fabrication is also often more cost-effective and scalable than conventional high-vacuum deposition techniques, for example, via high-throughput roll-to-roll processing^16^ or materials-efficient maskless printing methods. These combined advantages not only facilitate the precise tailoring of materials on the nanoscale for specific applications but also enable the rapid exploration of vast parameter spaces, thereby leading to accelerated materials discovery and optimization.

In this perspective, we examine how liquid-phase processing can address challenges associated with emerging functional materials. We first establish a unified framework for the integration of emerging materials into solution-fabricated devices, detailing the critical steps of synthesis and deposition of nanoscale building blocks. We then explore how this approach can unlock the potential of selected materials systems, focusing on ongoing research directions in our laboratory, namely, on liquid-metal nanoparticles, colloidal intermetallic nanocrystals, phase-change tellurides, and amorphous chalcogenides. Finally, we provide an outlook on how solution-phase design can enable accelerated materials optimization and discovery, driving advanced device integration across length scales.

## 2 Roadmap for Liquid-Fabricated Devices

Solution-phase fabrication of functional devices is a multistep process, schematically depicted in Figure 1. This process involves four interconnected levels of integration: (i) precursors (i.e., starting reagents), (ii) nanoscale building blocks, (iii) active layer, and (iv) final device architecture. Each level is crucial for achieving a functional device. Successful integration requires addressing unique challenges arising at the interfaces between the four levels:

(1) Precise synthesis of nanoscale building blocks (e.g., colloidal nanoparticles or molecular inks) converts starting chemicals into highly specific nanomaterials with preset properties, composition, and morphology.
(2) Controlled deposition of nanomaterials lays the foundation for devices, providing an active layer from assembled and sometimes annealed nanoscale building blocks.
(3) Finally, device fabrication completes the multilayer structure, involving nanofabrication steps compatible with liquid-deposited and often air-sensitive materials.

**Figure 1:**
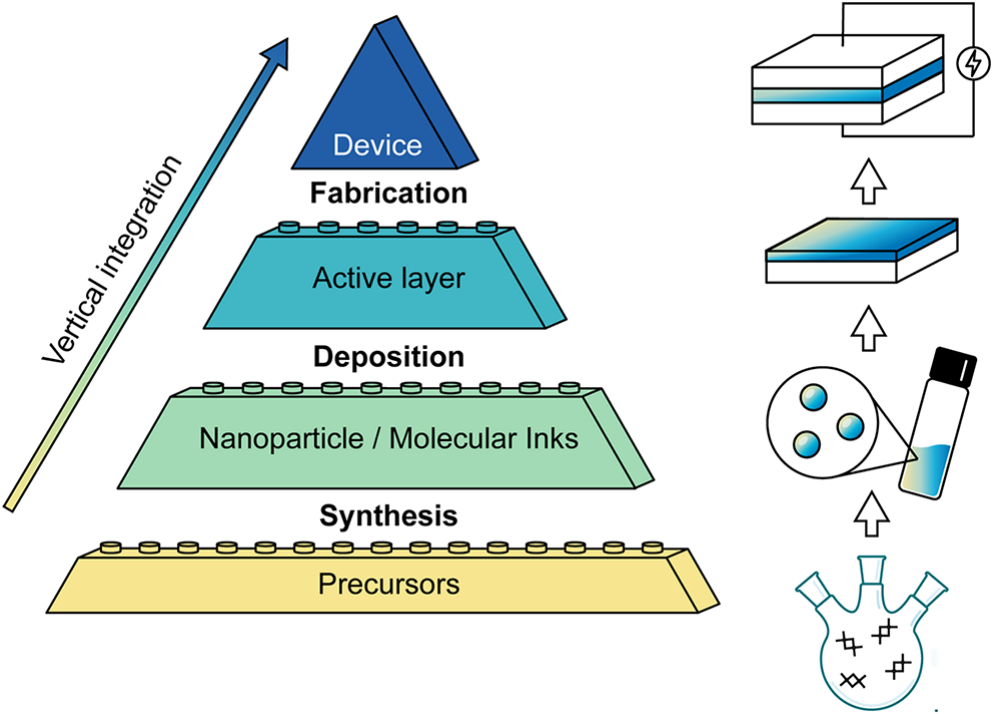
Roadmap of the liquid-phase fabrication of functional devices along the vertical integration axis, encompassing synthesis, deposition, and device fabrication.

These three steps bridge the four hierarchical levels of solution-engineered devices from wet chemical synthesis over materials and thin-film engineering to device science. Each subsequent level builds on the previous and provides further opportunities to optimize the nanomaterials to meet the quality bar of modern technologies. In this perspective, we focus on nanoscale building blocks and their deposition into the active layer of a device.

### 2.1 Nanoscale Building Blocks

Among liquid processable inks, colloidal nanoparticles and molecular chalcogenides stand out as particularly versatile building blocks, each offering distinct advantages for the controlled assembly of materials.

Colloidal nanoparticles leverage size-dependent properties for precise tuning of their properties, such as band gap, emission wavelength (Figure 2a),^17^ melting point, or crystallization temperature (Figure 2b–d).^18,19^ Nanoparticles from a wide range of materials (metals, chalcogenides, pnictides, oxides, and halides) can be prepared via hot injection or heat-up synthetic methods^18,20,21^ that enable precise control over size,^22^ composition,^23^ or shape.^24,25^ Further postsynthetic treatments such as annealing,^26^ shell growth,^27,28^ or ligand-exchange^29^ allow modification of defect density, surface passivation, band alignment, or charge carrier mobility. While significant progress in controlling and understanding every aspect of colloidal nanocrystals has been made, key challenges for colloidal liquid metals, intermetallics, or tellurides are the need for robust, scalable, and generalizable synthesis protocols and a deeper understanding of reaction mechanisms, the influence of synthesis parameters on the synthesis trajectory and final products, as well as possible intermediate and side products.^30^

**Figure 2:**
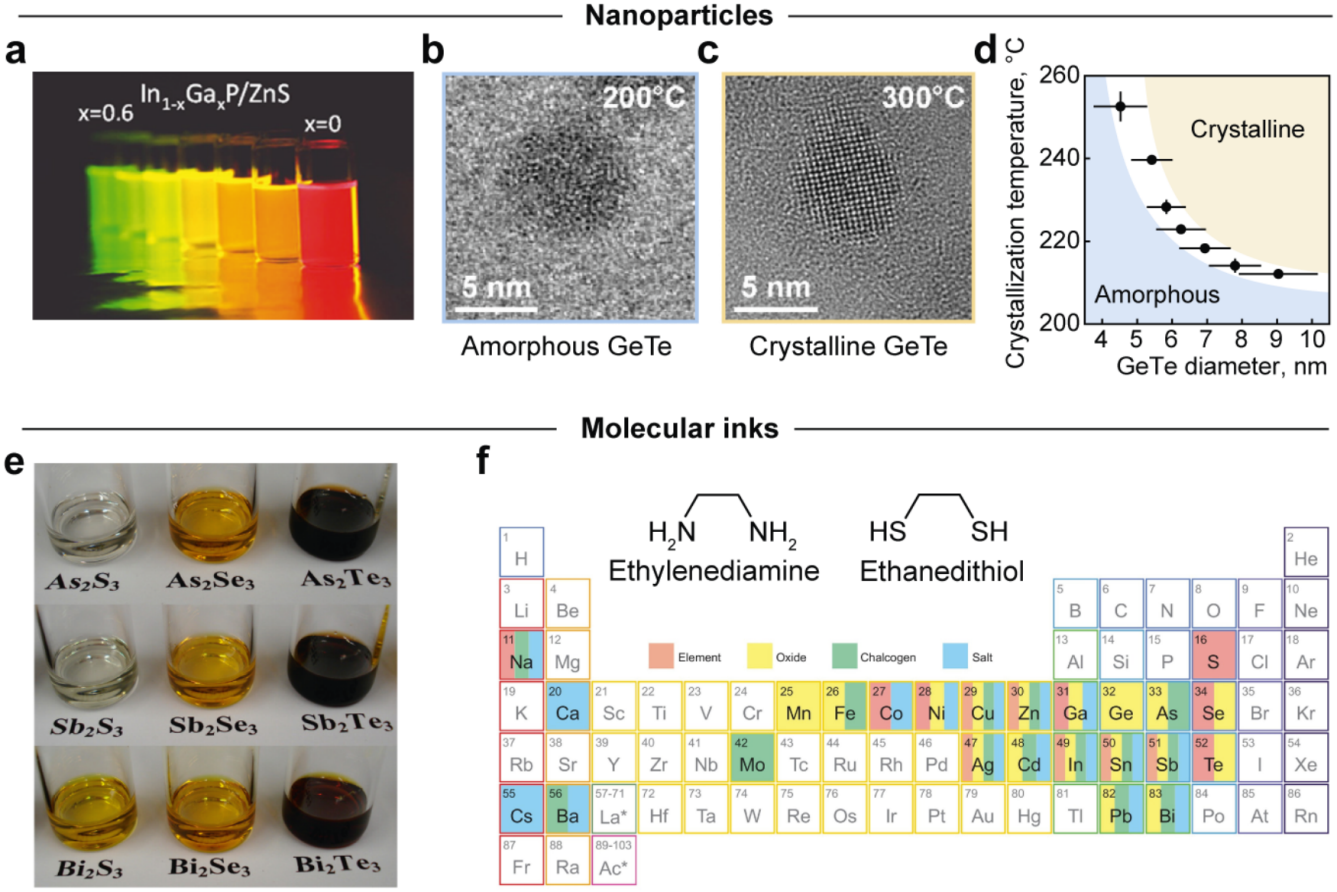
Solution processing of nanoscale building blocks. (a) Photograph displaying the range of colors of light emitted by core–shell In_1–*x*_Ga_*x*_P/ZnS samples with varying gallium content. (b, c) TEM micrographs of amorphous and crystalline GeTe nanoparticles and (d) size-dependent crystallization temperature. (e) Solutions of V–VI bulk chalcogenides in amine-thiol cosolvents. (f) Periodic table of elements soluble in amine-thiol cosolvents. The inset depicts the structural formulas of ethylenediamine and ethanedithiol. Panel (a) is reproduced from ref 28 Copyright 2023 American Chemical Society. Panels (b–d) are reproduced from ref 19 Copyright 2018 American Chemical Society. Panel (e) is reproduced from ref 37 Copyright 2013 American Chemical Society. Panel (f) is reprinted from Trends in Chemistry, Vol. 3, Koskela, K. M., Strumolo, M. J., and Brutchey, R. L., Progress of Thiol-Amine “Alkahest” Solutions for Thin Film Deposition, 1061–1073, Copyright 2021 with permission from Elsevier.

Molecular chalcogenide clusters and complexes are an alternative class of nanoscale building blocks^31,32^ that are typically smaller (<1 nm) than colloidal nanocrystals. These complexes require thermal annealing to vaporize the organics and crystallize the desired material. Their smaller size enables their assembly into dense thin films and structures with fewer voids, which is a significant advantage over nanocrystals, especially for electronic devices. Initially, they were made via dissolution of chalcogenide powders in hydrazine,^31^ which typically yields hydrazinium salts (N_2_H_5_^+^) of anionic metal-chalcogenide complexes. These can be composition-discrete (e.g., Sn_2_S_6_^4–^)^33^ or a mixture of different species (e.g., SbTe_3_^3–^, Sb_2_Te_5_^3–^, and Sb_2_Te_7_^4–^).^34^ Hydrazine-derived inks have been proven highly effective for the fabrication of thin-film transistors with high mobility (around 10 cm^2^ V^–1^ s^–1^ for SnS_2–_ *_x_* Se_*x*_)^3^ or high-efficiency chalcopyrite Cu­(In,Ga)­(S,Se)_2_ solar cells (>17%).^35^ Hydrazine is, however, toxic, explosive, and a suspected carcinogen,^36^ which limits industrial applications. As a result, more benign amine-thiol mixtures^32^ (mainly ethylenediamine and ethanedithiol) have emerged as a cosolvent, capable of dissolving various chalcogenides,^37^ chalcogens,^38^ oxides,^39^ and metals^40^ (Figure 2e,f), likewise forming molecular complexes with sizes below 1 nm.^37^ For some selenides and sulfides, the formation of metal-chalcogenide complexes (such as Sn_2_Se_6_^2–^)^41^ or metal thiolates^42^ is reported. Amine-thiol-derived materials are similarly highly performing, such as in chalcopyrite Cu­(In,Ga)­(S,Se)_2_, solar cells (16.39 and 17.1%, respectively, for amine-thiol and hydrazine-derived^35,43^ ) while also demonstrating high potential for applications in photocatalysis,^44^ resistive memory,^15,45^ or thermoelectrics.^46^

To improve the film quality and to reduce byproducts and impurities in solution-derived chalcogenides, several processing steps have been developed, including solvent engineering (i.e., replacing initial solvents under vacuum by precipitation in antisolvents, followed by redissolution)^41,47^ and chemical modification of dissolved species (e.g., adding elemental Se to replace sulfur in thiolate complexes^48^ or removing problematic polytelluride (Te_n_^2–^) chains through reduction and extraction).^46^

Despite these material-specific advances, a general protocol for molecular chalcogenide complexes is lacking, which highlights the need for a deeper understanding of chalcogenide species in solution, especially for highly promising tellurides. Several aspects remain unclear, starting from the dissolution chemistry in different amine and thiol solvents, potential rearrangements during extended dissolution and storage times, the exact effects of purification protocols, thermal decomposition pathways during annealing, and the origin and quantity of impurities (e.g., N, S, or O). Valuable insights into these processes are potentially gained by cluster characterization via mass spectrometry^49^ or nuclear magnetic resonance (NMR) under variation of amine and thiol solvents, dissolution times, or postsynthetic treatments. The decomposition pathways could be elucidated by combinations of thermogravimetry, mass spectrometry, infrared spectroscopy, and high-temperature X-ray diffraction. Understanding the elusive chemistry of molecular chalcogenide clusters will enable the reproducible preparation of pure materials, addressing device longevity bottlenecks and film-to-film variability. Such advances will ultimately mature the field of molecular inks from isolated, material-specific successes to generalized synthesis protocols of tailored chalcogenides for applications in thermoelectric, tunable photonics, and neuromorphic computing and fit for their integration in industrial-scale fabrication methods.

### 2.2 Liquid-Phase Deposition

Liquid-phase processing offers unparalleled flexibility in deposition methods, enabling the precise assembly of materials across length scales and geometries, which is crucial to translating the properties of these materials and integrating them into functional devices. For smooth and uniform materials layers, spin-coating^50^ is widely used to prepare thin films from molecular inks and nanocrystals and ranging from small substrates to wafer-scale deposition.^51^ It allows for precise control of the film thickness from several nanometers^33^ to the micrometer range^15^ by adjusting ink concentration, spin speed, and number of layers. For large-scale production, doctor blading, slot-die-coating, and spray coating are rapid and highly scalable methods to deposit thicker films (hundreds of nm to microns) and on large areas,^16,52^ well-suited for continuous high-volume roll-to-roll fabrication, such as for solar cells.^53^

Beyond thin films, liquid deposition also excels at creating patterned and 3D structures via additive manufacturing. Inkjet printing allows for direct, maskless patterning of materials in the micrometer range^54^ with resolution governed by ink rheology and droplet dynamics. Electrohydrodynamic printing uses an electric field to guide the deposition, achieving submicrometer pitch size and precision.^55^ 3D printing allows the additive manufacturing of intricate, three-dimensional structures, which are particularly attractive for thermoelectric devices.^56^

Finally, soft nanoimprint lithography provides a high-resolution stamping method for creating nanoscale patterns such as photonic arrays and metamaterials.^57,58^ Furthermore, it is also possible to infill small vias or holes as small as 50 nm by spin-coating or simple drop casting of nanocrystal or molecular inks.^15,59^ These methods open the avenue to applications that require nanoscale confinement, such as phase-change memory, photonics, and optoelectronic applications.

Thermal treatment is a crucial postdeposition step, which strongly impacts the final material structure and device performance by volatilization of organics, inducing crystal nucleation and growth as well as sintering of nanoscale building blocks. In nanocrystal thin films, soft baking at low temperatures suffices to remove residual solvents, preserving their small size and the associated quantum effects. Annealing at higher temperatures (>100 °C) can induce some extent of sintering, such as necking or even the formation of polycrystalline films, which, although it improves electronic transport properties by reducing grain boundaries, may lead to the loss of quantum confinement effects.^60^ A major challenge of nanocrystal films is porosity and voids, which can be detrimental to device longevity and performance.^61^ While a volume fraction of 74% represents the theoretical limit of perfect sphere packing, residual ligands and random packing can result in porosity far exceeding the theoretical estimate of 26%.^62^

In contrast, thin films from smaller molecular complexes are typically denser. They generally require higher annealing temperatures (200–550 °C) to form the desired materials by decomposition of molecular clusters. The final porosity of such films is, however, rarely quantified, but estimates from X-ray reflectivity fitting show that spin-coated SnS_2_ clusters can reach 90% of the bulk density,^63^ while a Sb_2_Te_3_ thin film was around 64%.^46^ Therefore, optimizing the density of ink-derived layers through optimizing synthesis, deposition, and annealing protocols holds significant promise to enhance the electrical performance and longevity of liquid-made devices to match the performance of high-vacuum-fabricated materials and ultimately leverage the opportunities of liquid-phase fabrication in an industrial context.

## 3 Emerging Materials

Many modern technologies were kick-started by singular breakthroughs in materials science,^64^ which are then often followed by lengthy cumulative advancements of materials chemistry, physics, and engineering to eventually achieve high performance and economically viable production. We argue that by leveraging the combined advantages of solution-phase processing and emerging materials, we can potentially compress these traditionally lengthy development cycles.

In this context, we focus on selected emerging nanomaterials, liquid-metal nanoparticles, colloidal intermetallics, multicomponent tellurides, and amorphous chalcogenides, whose potential is currently being unlocked by recent breakthroughs in liquid-phase processing. These materials are united by the common paradox of their unconventional properties, such as dynamic surfaces, metastable phases, and disordered structures, which are both the source of their rich functionalities and the reason they have been historically difficult to control. We highlight how solution-phase processing offers a powerful and versatile avenue to overcome these challenges by controlling these properties in the liquid phase, thereby providing new pathways for integrating these materials into next-generation devices.

### 3.1 Liquid-Metal Nanoparticles: Unique Properties Enable Rich Functionality

Liquid-metal nanoparticles, primarily based on gallium and its alloys, represent a class of materials with dynamic and unconventional chemical and physical properties. These attributes stem from Gallium’s unusual bonding,^7,65^ which results in a low melting point (*T*_m_ = 29.8 °C), volume expansion upon freezing, multiple (meta)­stable phases, an exceptionally high liquid range (up to 2304 °C), and a remarkably low vapor pressure.^66^ At the nanoscale, these effects are amplified by scaling, notably a pronounced melting point depression^67^ and an enhanced dynamic surface.^68^

These properties unlock a diverse range of properties. In catalysis, gallium nanoparticles can serve as hosts for other dissolved metals (e.g., Cu or Pd), which serve as highly active sites for ammonia synthesis or alkane dehydrogenation.^9,69^ The dynamic surface of the liquid metal makes them simultaneously resistant to coking or sintering, while the low vapor pressure prevents catalyst loss from evaporation during operation. In photonics, gallium nanoparticles also exhibit plasmonic resonances, tunable from the visible to the UV range (λ_LSPR_ = 800–240 nm) by controlling the particle size from 10 to 200 nm.^70^ The plasmonic resonance wavelength further varies between the solid and liquid state^71^ and crystal phases,^72^ making it attractive for tunable optical elements^71^ and optical data storage.^73^ In biomedicine, the low toxicity and physicochemical functionalities of Gallium nanoparticles offer promising functionalities for imaging, radiotherapy, and targeted drug delivery.^74^

The synthesis of liquid-metal nanoparticles is currently dominated by top-down and bottom-up approaches, with each offering distinct advantages. Top-down methods, such as ultrasonication^75^ or shear mixing^76^ (Figure 3a), directly fragment bulk metals like gallium or eutectic gallium–indium (eGaIn) into nanoparticles. These approaches are scalable (grams per batch) and fast (minutes to hours per batch). Major drawbacks are the broad size dispersions and limited size control (Figure 3b,c).^75^ Bottom-up colloidal synthesis, on the other hand, enables precise size control and narrow dispersity through thermolysis of molecular precursors (Ga_2_(NMe_2_)_6_ or GaCl_3_, see Figure 3d).^67,77^ This approach allows systematic tuning of particle size (12–46 nm) with narrow size distributions down to 7% relative polydispersity (Figure 3e,f). Despite its precision, the synthesis of gallium colloids exhibits unusual reaction trajectories, characterized by delayed nucleation onset, strongly overlapping nucleation and growth processes, and a tendency toward particle agglomeration during the synthesis. Achieving monodisperse gallium nanoparticles is therefore challenging, governed by the optimal gallium-to-amine ratio in the reaction mixture. To further balance the nucleation and growth kinetics, the process relies on adjusting synthesis parameters such as temperature and the chain length of the secondary amine.^78^

**Figure 3:**
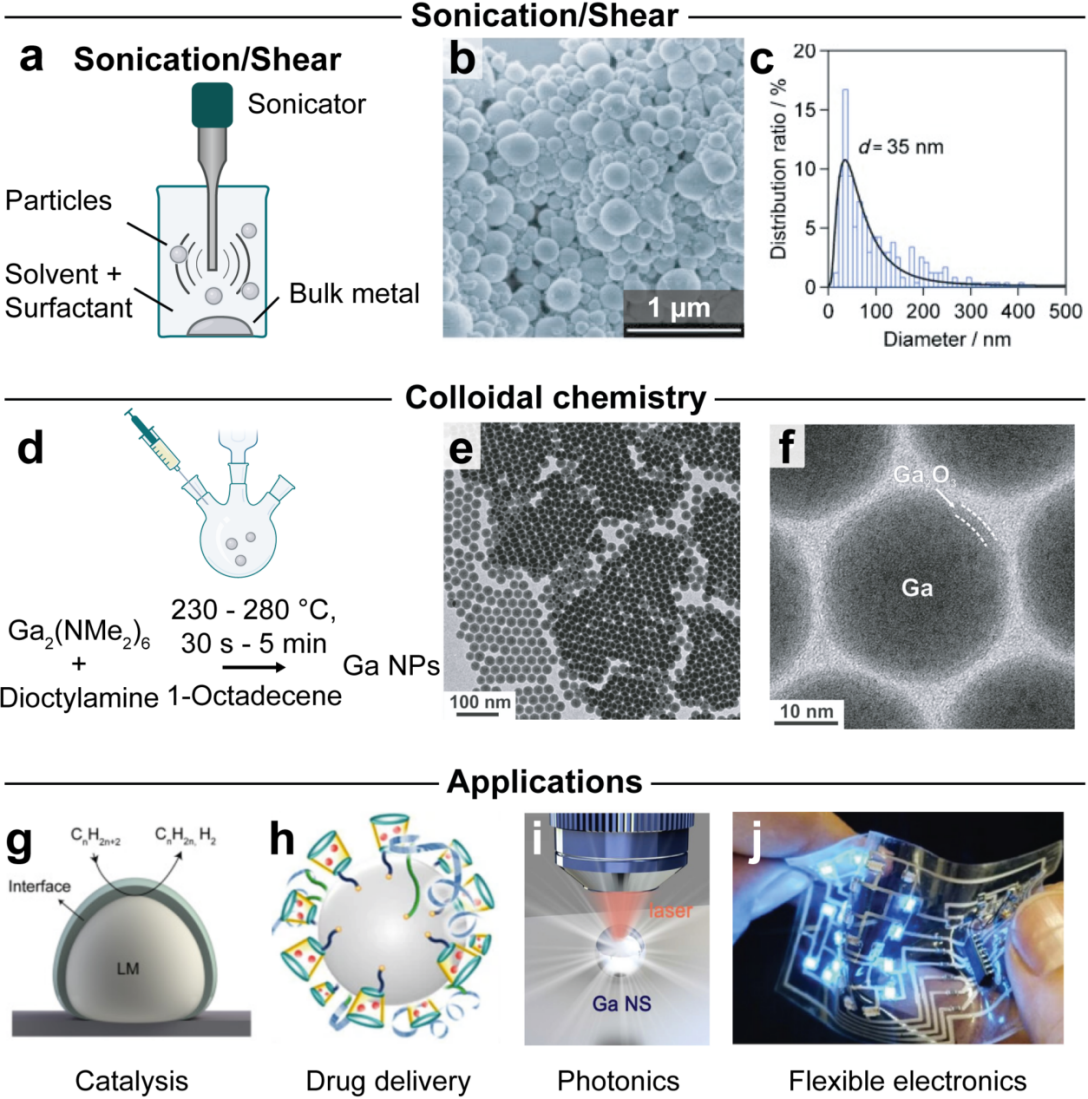
Synthesis and applications of liquid-metal nanoparticles. (a) Schematic depiction of the sonication process. (b) SEM micrograph and (c) size histogram of the resulting Ga particles.^83^ (d) Schematic depiction of the hot injection synthesis. (e) TEM micrograph of the resulting gallium nanoparticles with a size distribution as low as 7%. (f) High-resolution TEM showing a single Ga particle with a thin GaOx native shell.^75^ Examples of applications enabled by liquid-metal nanoparticles in (g) catalysis,^84^ (h) drug delivery, (i) photonics,^85^ and (j) flexible electronics.^82^ Panels (b, c) are reproduced with permission from ref 83. Panels (e, f) are reproduced from ref 75 Copyright 2018 American Chemical Society. Copyright 2015 Wiley-VCH. Panel (g) is reprinted from Joule, Vol. 4, Zuraiqi, K. et al., Liquid Metals in Catalysis for Energy Applications, p. 2290–2321. Copyright 2020, with permission from Elsevier. Panel (h) is reproduced from ref 86. Available under a CC-BY 4.0 license. Copyright 2015 Lu, Y. et al. Panel (i) is reproduced from ref 85 with permission from Wiley-VCH. Panel (j) is reproduced from ref 82. Available under a CC-BY 4.0 license. Copyright 2021 Lopes, P. A. et al.

Solution-phase synthesis also enables incorporation of other metals (i.e., Au, Ag, Pd, Cu, or In)^79,80^ to tune the materials functionality, for instance, for efficient electrocatalytic CO_2_ reduction. The naturally occurring gallium oxide shell can also be tuned in thickness through suitable surface ligands^68^ and can be functionalized for colloidal stability in aqueous media, targeted drug delivery applications, and to enhance its antimicrobial or anticancer properties.^2,81^

Further advances in liquid-metal nanoparticles will depend on the development of robust and scalable yet precise synthesis methods for this unconventional system. This requires a deep understanding of the underlying processes, but will enable the rational design of key materials properties such as their size, composition, and surface chemistry. This control will allow the tailoring of these materials for specific applications (Figure 3g–j), from tuning composition and surface properties for highly selective catalysts or embedding these nanoparticles within engineered solid-state or polymer matrices for flexible and self-healing electronics.^82^ Ultimately, this predictive approach will accelerate the discovery and integration of these dynamic materials into next-generation technologies.

### 3.2 Intermetallic Nanoparticles: Diversity by Design

Intermetallic and alloy nanoparticles combine two or more metals on the nanoscale and yield synergistic structural or electronic effects not found in single components. Intermetallic compounds possess unique crystal structures that lead to novel functionalities: Heusler alloys exhibit magnetic and spintronic functionalities, while skutterudites offer an enhanced thermoelectric performance. Highly ordered intermetallic phases (e.g., face-centered tetragonal-type PtFe)^87^ exhibit high catalytic activities, due to pronounced electronic effects enabling new reaction pathways and catalytic mechanisms. A new frontier in this field is high-entropy alloy nanoparticles, which contain more than four elements mixed in a uniform solid solution.^88^ They promise not only fine-tuning of catalytic activity and selectivity but also structural stability under harsh conditions.

Despite the immense potential of intermetallics through their structural and compositional diversity,^89^ the library of reported colloidal nanocrystals remains very limited. Synthesis challenges arise from combining two or more different metals with contrasting reduction potentials, oxophilicities, kinetics, and surface properties.^10^ If pronounced, these differences can lead to phase separation, nonstoichiometric compositions, or irregular size distribution. Consequently, despite case-specific successes for systems such as PtFe or CoPt_3_, a generalized synthesis route to homogeneous, monodisperse, intermetallic nanocrystals remains lacking.

Common colloidal synthesis strategies include coreduction, galvanic replacement, and seed-mediated growth.^90^ Coreduction is a straightforward one-pot approach where metal precursors are reduced in the presence of a coordinating solvent or capping ligands. This one-pot approach is generally fast and provides high reaction yields.^91^ However, the coreduction approach lacks universal applicability, particularly for metals with strongly different reduction potentials, resulting in phase separation or nonuniform compositions of intermetallic nanoparticles due to imbalanced nucleation of different metals. Furthermore, metal–ligand interactions vary significantly between different metals, making it necessary to tailor each system separately to achieve controlled growth of intermetallic nanocrystals.^10^

Seed-mediated approaches offer more control by separating nucleation and growth. Here, presynthesized monometallic seeds serve as a template for the second metal, introduced via a diffusive or galvanic step. This leverages the existing library of high-quality, single-metal nanocrystals of different sizes and shapes that have already been reported. Recently, the nanoscale amalgamation reaction offers a more generalized approach by combining presynthesized seeds (e.g., Ni, Pd, Cu, Ag, or Au nanocrystals) with a liquid metal (e.g., Ga, In, or Zn), dispatched to the seed surface by thermal decomposition of suitable precursors.^92^ This strategy enabled the synthesis of a wide variety of new intermetallics, even from highly dissimilar pairs of metals such as silver and gallium (Figure 4a–c). The nanoscale amalgamation reaction is a generalizable synthetic platform as long as one metal is liquid at the reaction temperature (typically 260–320 °C). Galvanic exchange is another common method where a metal salt is added to presynthesized metallic seeds.^93^ The metal ion with the higher reduction potential is reduced, while the other metal dissolves. The composition of intermetallic nanocrystals can be conveniently tuned by adjusting the amount of added metal salt. Furthermore, galvanic exchange can also proceed in the presence of a thin native oxide layer, as demonstrated for Sn/SnO_2_ ^94^ or gallium nanoparticles,^80,95^ allowing the facile introduction of In, Ag, or Cu.

**Figure 4:**
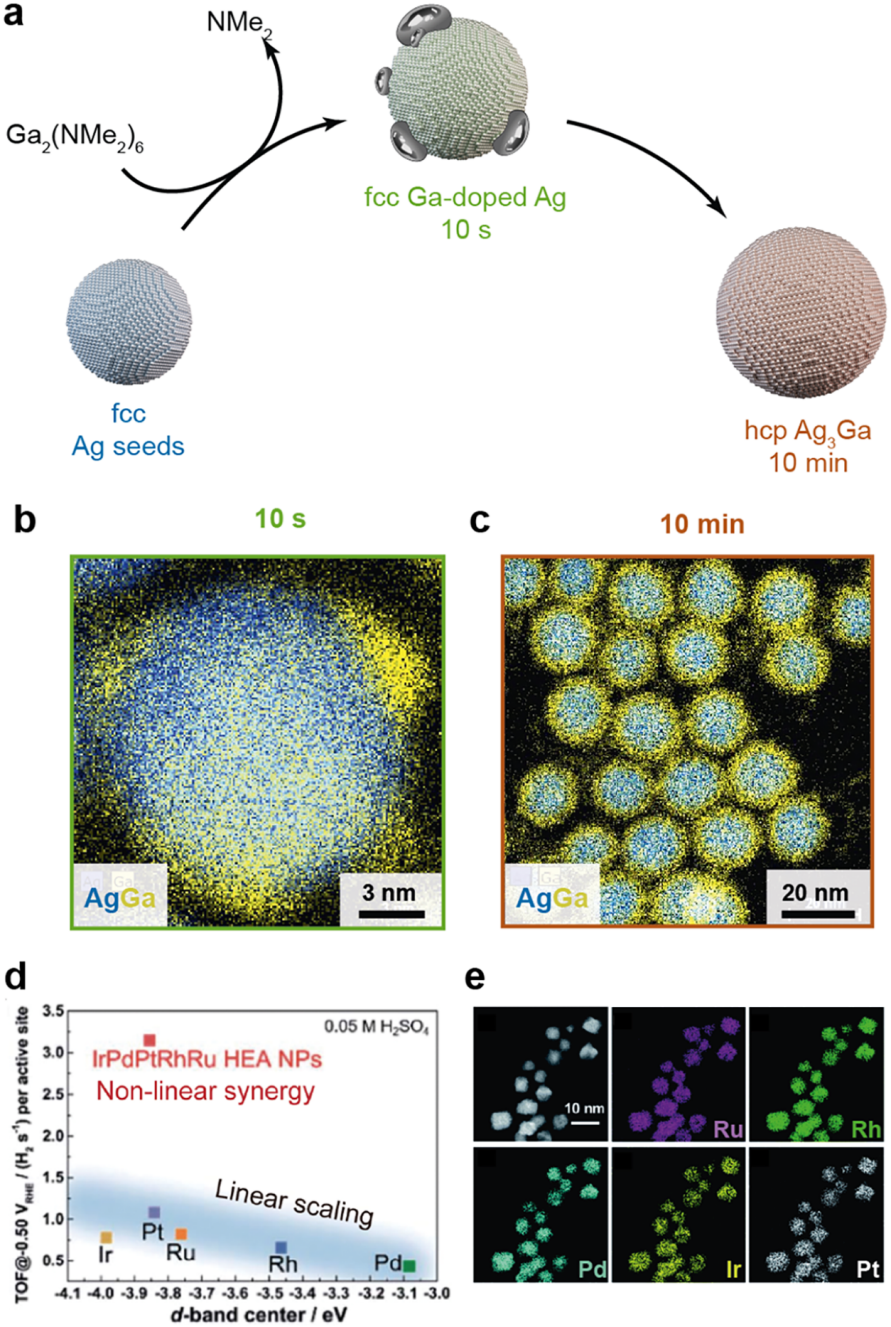
From intermetallic to high-entropy alloy nanoparticles. (a) Reaction scheme for the nanoscale amalgamation synthesis and (b, c) STEM-EDX maps of intermediate Ag/Ga structures and final Ag3Ga intermetallic nanocrystals. (d) IrPdPtRhRu HEA nanoparticles exhibiting a much higher turnover frequency in the hydrogen evolution reaction than that of the individual metals after a linear scaling relation. (e) HAADF-STEM image of IrPdPtRhRu HEA nanoparticles and corresponding EDX maps showing Ru-L, Rh-L, Pd-L, Ir-L, and Pt-L. Panels (a–c) are reproduced from Clarysse et al., Science Advances 10.1126/sciadv.abg1934 2021, AAAS. Panels (d, e) reproduced from ref 98. Available under a CC-BY 3.0 license. Copyright 2020 Wu, D. et al.

Despite these advances, the complexity of multielement systems means that each composition may have a unique reaction pathway, making the development of generalizable synthesis methods difficult, particularly considering the current shift from binary and ternary intermetallics to high-entropy alloy nanocrystals (Figure 4d,e). Moving beyond empirical trial-and-error approaches to data-driven materials discovery is therefore necessary. We envision the combination of computational and empirical methods in a continuous feedback loop. First, robotic high-throughput screening platforms must be established to explore the vast parameter spaces in nanocrystal synthesis, systematically varying precursors, ligands, temperatures, and stoichiometries. A key challenge is integrating these platforms with the strict inert conditions required for most intermetallic syntheses. This also involves the coupling with rapid characterization techniques such as small-angle X-ray scattering for analysis of particle size and size distribution^96^ or X-ray fluorescence to determine composition. Finally, machine learning and predictive models are trained on these data sets to predict synthesis outcomes. This will enable a rational design of experiment and the targeted synthesis of high-quality intermetallic and alloy nanocrystals. Crucially, these efforts must be supported by advanced characterization techniques, such as atomic electron tomography to precisely reveal the local atomic structure^97^ or in situ techniques such as wide- and small-angle X-ray scattering that will uncover the real-time formation mechanisms of these nanomaterials.

This approach has the potential to drastically shorten the development cycle of new materials and will enable the targeted synthesis of materials, such as precisely designed catalysts with unprecedented performance, stability, and selectivity, or the next generation of quantum and spintronic devices.

### 3.3 Phase-Change Tellurides: Customizing Memory for Future Devices

Phase-change materials can be reversibly switched between crystalline and amorphous states through electrical or light pulses.^4^ These distinct states exhibit strongly contrasting properties in their electrical conductivity, optical absorption, or refractive index,^4^ enabling a range of applications such as nonvolatile data storage and processing,^4^ brain-like neuromorphic computing,^99^ nonvolatile reflective displays,^100^ or programmable photonic elements.^101^

These properties are mainly found in metal tellurides, particularly those in the germanium–antimony–tellurium (GST, Figure 5a) system. Critically, the performance metrics of phase-change materials such as the crystallization temperature^102^ or switching speed^103^ are closely tied to their composition (Figure 5a,b), structure, and crystallographic orientation. For instance, Ge-rich GST (Ge_*x*_Sb_2_Te_5_ with *x* > 2) exhibits high crystallization temperatures (>300 °C)^104^ and thus improved thermal stability, making it particularly suitable for embedded memory applications in automotive and industrial circuits. This stability, however, comes at the expense of longer programming times compared to stoichiometric GST225 (>1 μs and 80 ns, respectively). In contrast, Sb_2_Te_3_-rich compositions exhibit faster switching speeds at the cost of lower crystallization temperatures (ca. 95 °C for pure Sb_2_Te_3_).^105^ Thus, these materials are favored in memory-oriented applications or neuro-inspired computing.^102^ Doping Sb_2_Te_3_ with Sc, Y, or Ti can overcome this inherent speed/stability trade-off. For instance, scandium-doped Sb_2_Te_3_ shows switching speeds below 1 ns and simultaneously, increased crystallization temperature (170 °C).^106^

**Figure 5:**
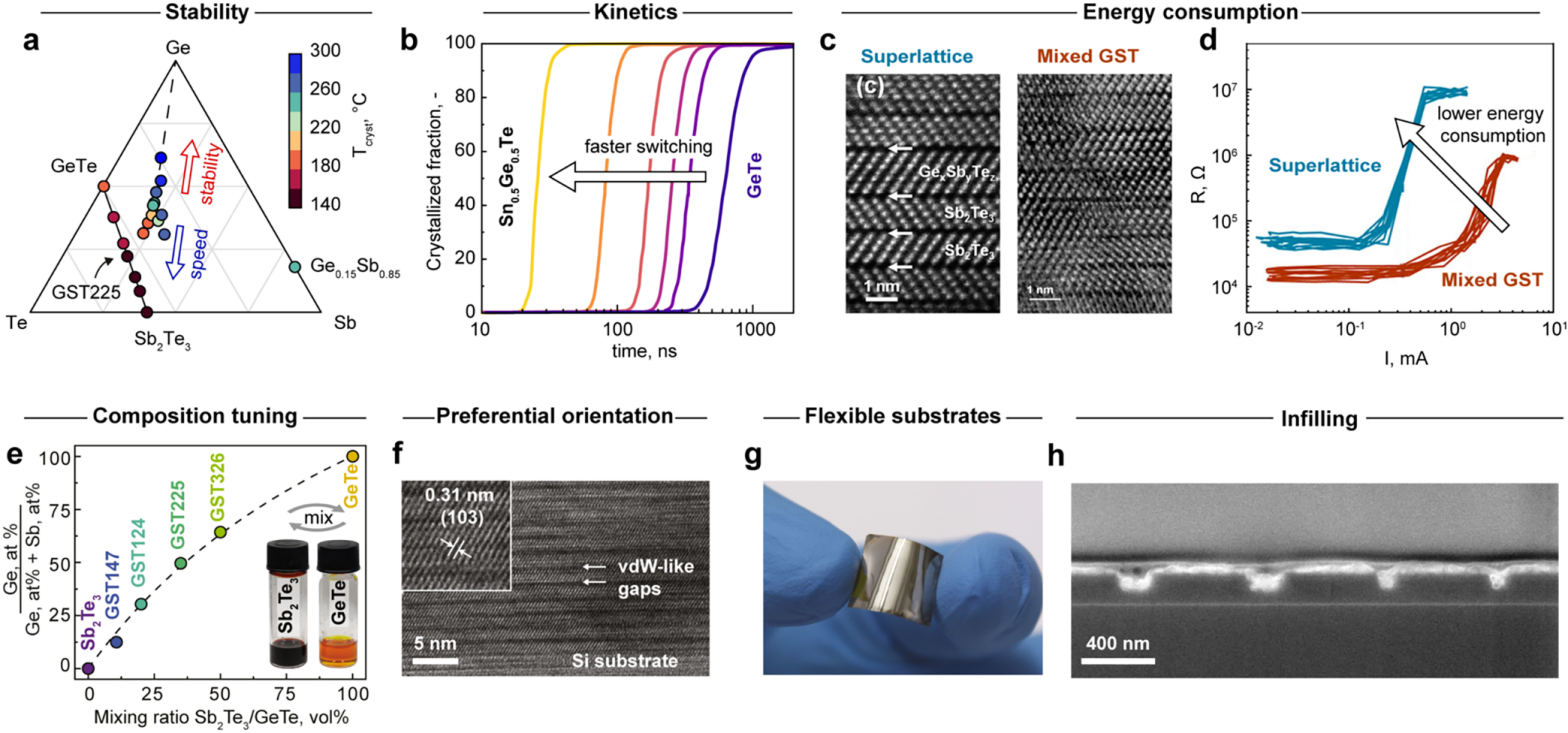
Tuning Phase-Change Tellurides through composition, structure, and deposition. (a) Crystallization temperature as a function of composition in the ternary Ge–Sb–Te phase diagram. The solid black line indicates compositions along the stoichiometric pseudobinary GeTe-Sb2Te3 system, while the dashed black line indicates Ge-rich GST. (b) Crystallization probability as a function of time and composition for GeTe-SnTe alloys. (c) HAADF-STEM images of a Sb2Te3/GexSbyTez superlattice and mixed GST with van der Waals-like gaps indicated.^6^ (d) Resistance as a function of the programming current for superlattice versus mixed GST phase-change memory devices. (e) Ge content in solution-processed GST thin films as a function of mixing ratio between GeTe and Sb2Te3 inks. The inset shows images of the Sb2Te3 and GeTe molecular inks. (f) HR-TEM image of the ink-deposited GST225 thin film, highlighting high crystallinity and strong texture in the (00l) direction. Inset measures lattice fringes with a distance of 0.31 nm, corresponding to (103) planes. (g) GST225 film spin-coated on a flexible polyimide substrate. (h) Cross-sectional SEM micrograph of nanoscale trenches infilled with solution-processed GST225. Panel (a) is replotted from ref 102. Available under a CC-BY 4.0 license. Copyright 2021 Sun, L. et al. Panel (b) is replotted from ref 103. Available under a CC-BY 4.0 license. Copyright 2021 Persch, C. et al. Panels (c, d) are reproduced from ref 8 Copyright 2022 American Chemical Society. Panels (e–f) are reproduced from ref 15 Copyright 2023 American Chemical Society.

The anisotropic structure of tellurides with layered arrangements and van der Waals-like gaps can also be leveraged to tune memory performance. For instance, the thermal conductivity is 60% lower across the plane compared to the in-plane direction for GST.^107^ This limits thermal dissipation and lowers the energy consumption in highly textured GST memory devices. This concept is also exploited in superlattice-based phase-change memories, where ultrathin layers of different materials (e.g., 2 nm Sb_2_Te_3_ and 1.8 nm GST, Figure 5c) are stacked sequentially. The increased number of interfaces leads to notably lower energy consumption compared to GST-only devices (Figure 5d).^8^ Alternatively, an anisotropic thermal barrier can also be introduced by Ti-doping of Sb_2_Te_3_.^108^ TiTe_2_ forms distinct layers within Sb_2_Te_3_ due to its immiscibility, thereby lowering thermal conductivity and increasing crystallization speed.

Phase-change telluride thin films are typically fabricated via high-vacuum deposition methods,^109^ such as sputtering (or PVD, physical vapor deposition), chemical vapor deposition (CVD), or atomic layer deposition (ALD). While effective for producing uniform films, these techniques can be costly, energy-intensive, and limited in their ability to flexibly vary materials parameters. Liquid deposition of phase-change materials at ambient pressure offers a low-cost, scalable, and highly flexible alternative to high-vacuum processes.^110^ The dissolution of bulk tellurides in hydrazine or amine-thiol cosolvents^32,37^ yields molecular inks of a wide variety of phase-change materials such as GeTe, Sb_2_Te_3_, Sc_2_Te_3_, or TiTe_2_.^15,46^ Composition variation through simple mixing of inks gives access to prototypical Ge–Sb–Te or modern Sc- or Ti-doped Sb_2_Te_3_ materials, as shown in Figure 5e–h. For device fabrication, liquid deposition offers the conformal infilling of nanoscale device structures and deposition on flexible or curved substrates. Molecular inks are also compatible with a vast variety of deposition methods, from 3D printing to soft nanoimprint lithography (see Figure 6). This has the potential to enable novel memory architectures, such as high-aspect ratio devices, and scalable low-cost fabrication of tunable metasurfaces and phase-change nonvolatile displays.

**Figure 6:**
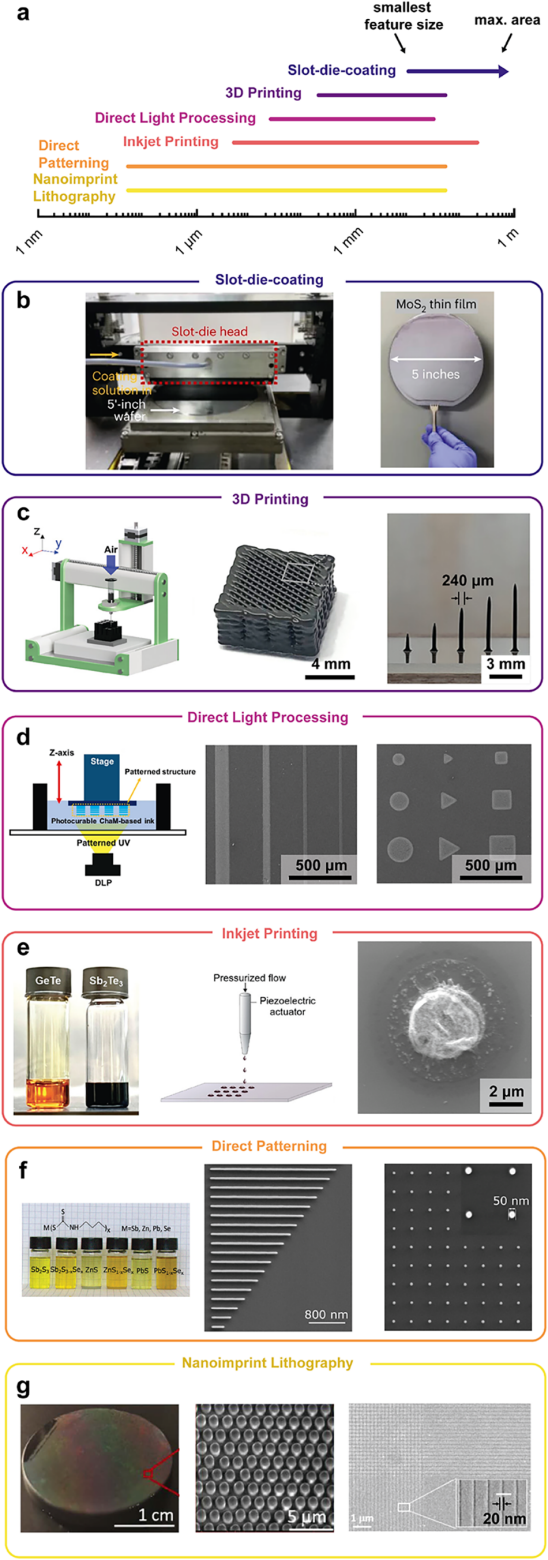
Patterning of solution-processed functional chalcogenides across length scales. (a) Comparison of length scales for different patterning methods. The lines reach from the minimum feature size to the maximum deposited area and are extracted from the references below. (b) Wafer-scale slot-die coating of MoS_2_ nanosheets.^125^ Kwon, Y. A. et al., Nature Electronics, Vol. 6, Springer Nature, 2023, reproduced with permission from SNCSC. (c) Extrusion-based 3D printing of silver chalcogenide microparticles. Reprinted from ref 56. Available under a CC-BY 4.0 license. Copyright 2024 Kim et al. (d) Direct light processing of metal-chalcogenide inks, UV-sensitized with a photoacid. Reprinted from ref 127. Available under a CC-BY 4.0 license. Copyright 2024 Baek, S. et al. (e) Inkjet printing of Ge–Sb–Te derived from amine-thiol-based inks. Reprinted from ref 126. Available under a CC-BY 4.0 license. Copyright 2024 He et al. (f) Direct patterning of chalcogenides via lithography of metal-butyldithiocarbamic acids that act as negative resists. Reprinted from ref 128. Available under a CC-BY 4.0 license. Copyright 2020 Wang W. et al. (g) Nanoimprint lithography of solution-processed As_2_Se_3_ thin films. Reprinted with permission from ref 58 Copyright 2020 Optical Society of America.

Molecular inks also have the potential to significantly accelerate materials discovery by enabling combinatorial synthesis to explore the vast compositional spaces of phase-change materials. Automated systems, such as liquid dispensers or inkjet printers, can create hundreds of different compositions on a single chip. These can then be characterized via high-throughput techniques, e.g., by laser switching experiments to deduce crystallization power and kinetics without the need for full device integration.^103^ This approach will facilitate the rapid discovery of new materials and fine-tuning of their properties. Critically, these findings can be directly implemented in traditional high-vacuum deposition methods and thus can benefit the entire field.

As a complementary approach, colloidal nanoparticles of phase-change tellurides represent an excellent experimental platform for fundamental studies. For instance, GeTe^19,111^ nanoparticles can be produced with fine control over the size and size uniformity, revealing a size-dependent crystallization temperature for sub-10 nm GeTe colloids (see Figure 2b–d). A library of ternary M–Ge-Te (M = Sn, Pb, In, Bi, Ag, Co, or Cu)^112,113^ nanoparticles has also been reported with excellent composition tuning. Therefore, phase-changed memory nanoparticles can be seen as model systems, for which memory properties can be tuned independently by composition and size. This offers new avenues to overcome the speed/stability trade-off in bulk tellurides and to design scaling rules for ultimately small-sized phase-change memory devices.

### 3.4 Glassy Chalcogenides: Taming Disorder to Unlock Seamless Functionality

Chalcogenide glasses (ChGs) are amorphous materials based on sulfur, selenium, or tellurium compounds. Their nonequilibrium disordered structure, coupled with the absence of grain boundaries and the ability to finely tune composition, unlocks unique functionalities and opportunities to design application-specific materials. For optoelectronics, chalcogenide glasses are promising for optical fibers and waveguides due to their optical transparency extending from the visible to the mid-infrared (IR) region, coupled with high refractive indices.^114^ They also possess exceptionally high nonlinear optical coefficients, critical for ultrafast all-optical signal processing, switching, and supercontinuum generation.^115^ For data storage and neuromorphic computing, ChGs exhibit nonlinear electrical properties that are typically exploited in ovonic threshold switches (OTS).^116^ OTS devices leverage the rapid and repeatable transition of amorphous chalcogenides from a highly resistive to a highly conductive state upon exceeding a threshold voltage. Remarkably, this electronic switching mechanism is nondestructive, making OTS ideal as selector elements in high-density, low-footprint 1S1R (one selector one resistor) memory cells, as also realized in commercialized phase-change random access memory (e.g., Intel/Micron’s 3D XPoint technology).^116^ The threshold voltage scales with film thickness and depends on the band gap, thus allowing for the tailoring of the selector characteristics.^117^ Finally, the versatility of ChGs is further highlighted by their use as highly conductive and stable solid-state battery electrode materials^118^ or efficient and stable photocatalysts (MoS_2_).^119^

Traditionally, the fabrication of ChG thin films for both optical and electronic applications relies heavily on high-vacuum deposition methods like magnetron sputtering,^117^ physical vapor deposition (PVD), or atomic layer deposition (ALD).^120^ While these techniques offer excellent control over film thickness and composition, they present limitations in terms of cost, scalability, energy consumption, and infilling high-aspect ratio features. In contrast, solution-phase processing of chalcogenide glasses has emerged as a promising alternative, explored since the early 1980s with the dissolution of As_2_S_3_ in amines.^121^ This versatile approach has since been extended to chalcogenide-rich germanium glasses (e.g., GeSe_2_) and more complex ternary compositions (e.g., Ge_23_Sb_7_S_70_).^122^ These thin films show remarkable surface quality (i.e., RMS roughness of sub-1 nm) and optical properties comparable to evaporated samples, yet no electronic properties or applications have been reported to date.

The integration of chalcogenide glasses in the form of molecular inks or colloidal nanoparticles represents a powerful platform to advance the field of chalcogenide glasses. The solution-phase approach is highly attractive to enable novel device architectures such as 3D structures or flexible electronics. Furthermore, it can facilitate the exploration of structure–function relationships of amorphous materials or accelerate the discovery of new materials via combinatorial synthesis of multinary compositions. Finally, it is an excellent experimental platform for designing scaling rules for ultrasmall devices using colloidal nanoparticles.^19,123^

## 4 Outlook and Future Trends

Emerging materials such as liquid metals, intermetallics, phase-change materials, and glassy chalcogenides offer unconventional properties that promise innovations in catalysis, biomedicine, data storage, or neuromorphic computing. However, the unique characteristics, such as dynamic surfaces, complex bonding, and metastable or amorphous phases, enable the unique functionalities and also present challenges to control and tailor emerging materials for their specific application. Solution-phase assembly of materials from nanoscale building blocks offers a powerful avenue to overcoming these barriers. To fully realize this promise, the field must advance at two parallel yet interconnected frontiers.

The first frontier is to build a deep understanding of the individual processes that occur during synthesis and deposition. The intrinsic challenges of emerging materials, such as mismatched component reactivity, complex dissolution, and transient decomposition processes, must be overcome in order to move beyond materials-specific successes toward robust synthesis protocols for nanocrystals and molecular clusters across the periodic table. In this context, recent advancements in in situ and ex situ characterization techniques to elucidate complex reaction and assembly mechanisms in solution are becoming increasingly promising. Methods such as small-angle and wide-angle X-ray scattering or total X-ray scattering are critical tools to understand the influence of parameters such as time, temperature, or the choice of precursors. Mass spectrometry or nuclear magnetic resonance is used to shed light on the cluster chemistry of molecular chalcogenides, while monitoring their decomposition can be studied via in situ annealing infrared spectroscopy, X-ray diffraction, and thermogravimetric analysis. Understanding these processes will enable rational materials design, moving the synthesis of advanced solution-based materials from empirical trial-and-error to a generalizable experimental platform. Solution-based approaches are also particularly well-suited for high-throughput data generation, serving as input to design-of-experiment algorithms or closed-loop machine learning-driven workflows,^124^ thus driving the discovery of new functional materials and the optimization of existing ones at accelerated speeds.

The second frontier is to harness the existing power of solution-based techniques to drive material integration across diverse length scales and enable rapid prototyping due to their compatibility with a variety of advanced patterning methods. Figure 6a shows an overview of this research direction. For industrial-scale manufacturing, roll-to-roll processing via slot-die coating^125^ or spray coating^41^ is particularly advantageous due to high cost-effectiveness, scalability, and ability to operate at ambient environments (Figure 6b). Additive manufacturing (Figure 6c) allows targeted geometrical design of complex device structures, which is particularly attractive for thermoelectric waste heat regeneration, where specific architectures significantly enhance performance.^56^ Inkjet printing (Figure 6e) provides cost-effective and maskless patterning, and enables rapid prototyping and fabrication of devices such as phase-change memories.^126^ For nanoelectronic devices requiring high precision, advanced patterning can be achieved using photosensitized metal-chalcogenide inks and a commercial-grade DLP printer^127^ or patterning metal chalcogenides via direct lithography (Figure 6e,f).^128^ Scalable production of intricate nanostructures or metamaterials can also be achieved via soft nanoimprint lithography (Figure 6g).^58^ These diverse capabilities underscore how solution processing can facilitate the translation of novel material discovery to functional and scalable device fabrication.

By pursuing these two synergistic paths, solution-phase processing will evolve from a specialized synthetic tool into a cornerstone of modern materials innovation, drastically shortening the development cycle of emerging materials to commercial viability. More broadly, the unparalleled flexibility of solution-phase processing in manipulating composition, structure, and geometry offers transformative potential to control emerging materials and their properties.
